# Predicting the Ultimate Axial Capacity of Uniaxially Loaded CFST Columns Using Multiphysics Artificial Intelligence

**DOI:** 10.3390/ma15010039

**Published:** 2021-12-22

**Authors:** Sangeen Khan, Mohsin Ali Khan, Adeel Zafar, Muhammad Faisal Javed, Fahid Aslam, Muhammad Ali Musarat, Nikolai Ivanovich Vatin

**Affiliations:** 1 Department of Structural Engineering, Military College of Engineering (MCE), National University of Science and Technology (NUST), Islamabad 44000, Pakistan; sakhan.pg18mce@student.nust.edu.pk (S.K.); adeel.zafar@mce.nust.edu.pk (A.Z.); 2 Civil Engineering Department, CECOS University of IT and Emerging Science, Peshawar 25000, Pakistan; 3 Department of Civil Engineering, COMSATS University Islamabad, Abbottabad Campus, Abbottabad 22060, Pakistan; 4 Department of Civil Engineering, College of Engineering in Al-Kharj, Prince Sattam bin Abdulaziz University, Al-Kharj 11942, Saudi Arabia; f.aslam@psau.edu.sa; 5 Department of Civil and Environmental Engineering, University Teknologi PETRONAS, Bandar Seri Iskandar 32610, Malaysia; muhammad_19000316@utp.edu.my; 6 Peter the Great St. Petersburg Polytechnic University, 195291 St. Petersburg, Russia; vatin@mail.ru

**Keywords:** concrete filled steel tube, artificial neural network, multi-physics model, Random Forest Regression, Adaptive Neuro-Fuzzy Inference System, gene expression programming, bearing capacity of columns

## Abstract

The object of this research is concrete-filled steel tubes (CFST). The article aimed to develop a prediction Multiphysics model for the circular CFST column by using the Artificial Neural Network (ANN), the Adaptive Neuro-Fuzzy Inference System (ANFIS) and the Gene Expression Program (GEP). The database for this study contains 1667 datapoints in which 702 are short CFST columns and 965 are long CFST columns. The input parameters are the geometric dimensions of the structural elements of the column and the mechanical properties of materials. The target parameters are the bearing capacity of columns, which determines their life cycle. A Multiphysics model was developed, and various statistical checks were applied using the three artificial intelligence techniques mentioned above. Parametric and sensitivity analyses were also performed on both short and long GEP models. The overall performance of the GEP model was better than the ANN and ANFIS models, and the prediction values of the GEP model were near actual values. The *PI* of the predicted *N_st_* by GEP, ANN and ANFIS for training are 0.0416, 0.1423, and 0.1016, respectively, and for *N_lg_* these values are 0.1169, 0.2990 and 0.1542, respectively. Corresponding *OF* values are 0.2300, 0.1200, and 0.090 for *N_st_*, and 0.1000, 0.2700, and 0.1500 for *N_lg_*. The superiority of the GEP method to the other techniques can be seen from the fact that the GEP technique provides suitable connections based on practical experimental work and does not rely on prior solutions. It is concluded that the GEP model can be used to predict the bearing capacity of circular CFST columns to avoid any laborious and time-consuming experimental work. It is also recommended that further research should be performed on the data to develop a prediction equation using other techniques such as Random Forest Regression and Multi Expression Program.

## 1. Introduction

### 1.1. Concrete Filled Steel Tube Artificial Modelling

Concrete filled steel tube (CFST) is a composite construction element. CFST columns are advantageous due to greater seismic resistance and load bearing capacity, lesser size utilization, good aesthetics, and high fire resistance [1]. The composite action of steel tube and infilled concrete improves the overall strength and ductility of CFST columns. CFST construction does not require concrete casting formwork, which helps in faster construction with lesser cost [2]. Different types of CFST cross-sections are used in the building industry, such as elliptical, square, rectangular, polygonal, and circular sections. However, this research is concerned with the circular CFST stub and long columns. Generally, the structural performance of circular CFST columns is better than that of polygonal CFST columns [3]. Circular CFST columns have higher ultimate capacities as compared to other cross-sectional shapes. The strength index value for the circular column is higher than 1, which shows the positive confinement effect of the circular CFST column. The rectangular CFST column has less confinement effect, and thus it can be seen from the strength index that the theoretical capacity is greater than the actual capacity [4].

Numerous researchers have conducted experimental studies to check the effect of different parameters on the axial capacity of circular short and long CFST columns [5]. These parameters include concrete compressive strength, diameter, height of the column, yield strength of steel tube, thickness of steel tube, and eccentricity. Furthermore, the experimental research is costly and needs expensive precise equipment. The accuracy of the experimental study relies on skilled labor, type of equipment, condition for casting and testing of specimens and appropriate instrumentation, while in numerical or analytical modelling high computational skills are necessary and need experimental tests merely to validate the model. Thus, developing an accurate, precise, and reliable empirical expression is essential to encompass all the important parameters. The short columns are described as those with *L/D* ≤ 4 (for circular columns) or *L/B* ≤ 4 (for rectangular columns), where L is the length of the specimens, and D and B represent the outer diameter of the circular section the width of the rectangular sections respectively, slender columns having *L/B* > 4 or *L/D* > 4 [6].

CFST columns have been used for operation in extreme conditions under high axial loads. High loads require greater dimensions of the CFST cross-section, high strength steel tube and concrete. The dimensions and strength of the material are limited in practice, and these empirically determined limitations are included in building codes. Each code differently interprets the effect of confinement on the overall bearing capacity of CFST columns. Chinese code [7] and Japanese code [8] use allowable concrete strength (*f_c_′*) of 67 MPa and 90 MPa and steel tube yield stress (*f_y_*) of 420 MPa and 440 MPa, respectively. The recently released AISC 360-16 [9] and AS/NZS 2327 [10] is applicable to *f_c_′* of 69 MPa and 100 MPa and *f_y_* is limited to 525 MPa and 690 MPa, respectively. The Eurocode [11] permits *f_c_′* and *f_y_* up to 50 MPa and 460 MPa, respectively. In comparison, Liew and Xiong [12] extended these limits to 90 MPa and 460 MPa, respectively. The use of high strength material is valuable for reduction of the size of CFST columns which eventually leads to the savage of floor space and lesser construction cost. The high strength steel tube enhances the elastic behavior and thus improves the confinement effect towards the concrete core. Use of concrete in CFST helps in the functional optimization of both materials. Advancements in the construction industry permit high strength materials to be practically utilized. In addition, the equations available in the mentioned standard codes do not agree with each other. Moreover, these codes are based on the pre-assumed stress–strain curve of CFST, which makes the validity of the presented equations suspicious. To tackle this issue, many researchers have conducted experimental studies on the utilization of high strength materials in CFST columns. Khan et al. [13] use *f_c_′* and *f_y_* up to 113 MPa and 762 MPa, respectively, in CFST columns. Mursi and Uy [14] and Sakino et al. use normal strength concrete in CFST columns with *f_y_* up to 761 MPa and 853 MPa, respectively.

Different studies recommended various methods for the estimation of the bearing capacity of CFST columns [15]. Researchers have found nonlinear and linear regression methods to be highly effective in the civil engineering field [16]. However, the development of these models is based on pre-assumed equations, which makes them impracticable and unrealistic in terms of estimation perspective [17,18]. To tackle this problem, recently various artificial intelligence (AI) techniques, specifically machine learning methods, have been extensively used in the field of civil engineering) [19,20,21]. Researchers have used Artificial neural network (ANN) [22], Support vector machine (SVM), [23] random forest regression (RFR) [24,25], adaptive neuro-fuzzy interface system (ANFIS) [26], feed-forward neural network (FNN) [27], particle swarm optimization (PSO) [28], genetic programming (GP) [29], gene expression programming (GEP) [30], etc. For the estimation of mechanical properties of different types of civil engineering materials and structures. Nguyen et al. [31] estimated the axial capacity of rectangular CFST via the FNN algorithm. Researchers also projected the relationship between load deformation and fire resistance of CFST stub columns using the ANN technique [32].

Other studies [33] proposed an ANN model to estimate the ultimate capacity of rectangular CFST beam-columns and circular CFST beams. It was concluded that the predictive model performed better than EC4 and AISC projected equations in both cases. Likewise, the authors focused on an alternative technique to estimate the ultimate axial capacity of stub CFST columns and confinement performance of infilled concrete ANFIS [34,35]. They stated that the ANFIS model performs significantly better and is more accurate than multiple linear regression (MLR) and multiple non-linear regression (NLMR). The researchers also formulated the punching shear strength of concrete slabs using ANN and GP algorithms [36]. The GEP empirical model for predicting the axial capacity of short circular CFST columns performed better than other formulae available in different design codes [37]. Similarly, researchers use gene expression programming (GEP) to deliver an empirical equation for the estimation of the axial capacity of concrete filled double skin tube (CFDST) columns [38] and short CFST columns [39]. They testified that GEP predictive models yield better performance than available equations, giving lesser error values with a higher correlation coefficient.

Furthermore, the soft computing algorithms solve problems by training the available data set to obtain results, which are then validated via validation set data [40]. However, ANN and ANFIS algorithm-based prediction needs many improvements to provide a practical equation for future use. Therefore they are black boxes [41]. Numerous hidden neurons collapse ANN and ANFIS algorithms to provide a practicable empirical equation between the explanatory variables and the response, and can be adopted to predict correlation purposes [41]. The complex structure of these models obstructs wide-scale adoption [41]. However, they can be effectively utilized as a predictor and to judge the accuracy of the GEP based model [41].

**Figure 1:**
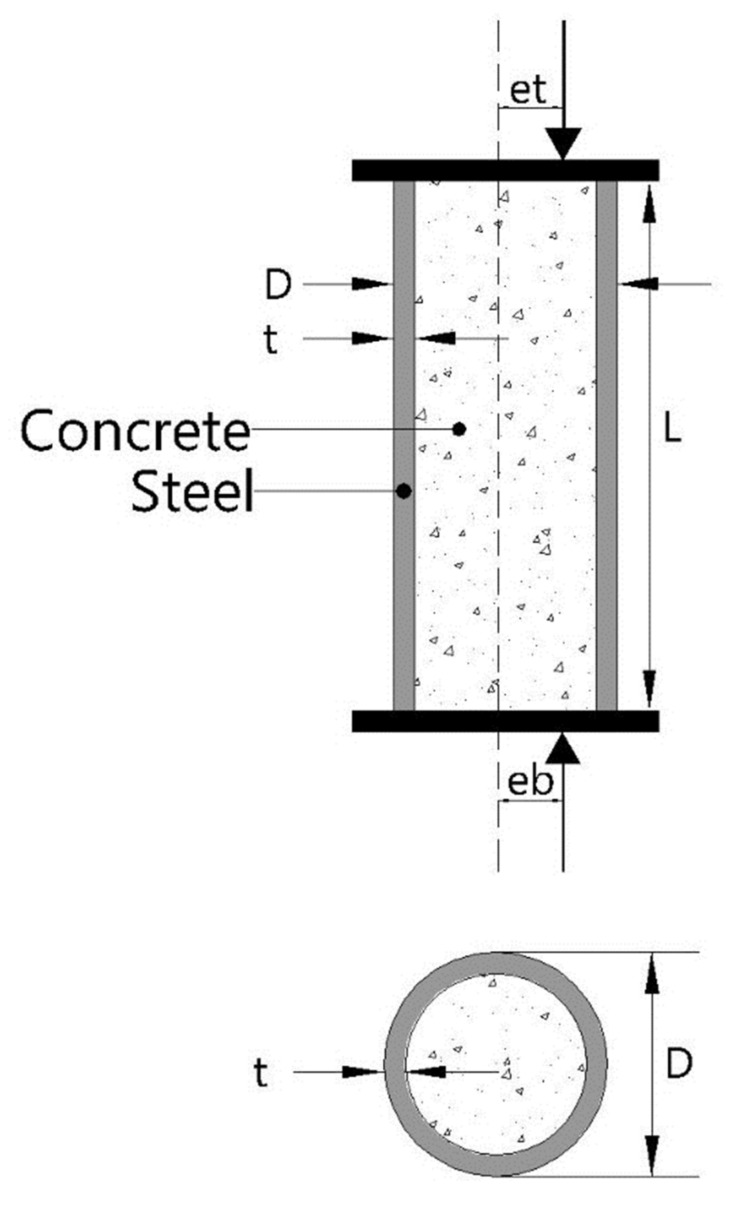
Schematic layout of circular concrete filled steel tube (CFST) column.

In addition, experimental research is costly and requires abundant resources and time to carry out an accurate strength analysis. A slight mistake in computing the quantities and casting process may cause a malicious impact on the strength. Besides, the machine learning algorithm only requires an initial data set to efficiently predict the desired property. In this research, an effort has been made to address the limitation of the provision of the standard code by developing a GEP based empirical equation for short and slender CFST beam columns considering several input variables, i.e., *D*: diameter of the tube, *t*: thickness of the tube, *L*: length of the tube, *L/D*: length to diameter ratio, *e_t_*: eccentricity at the top face or loading face, *e_b_*: eccentricity at the bottom face, *f_y_*: yield strength of the tube and *f_c_*: compressive strength of the infilled concrete. The schematic layout showing the input variables used to predict the capacity of CFST columns has been provided in Figure 1. The GEP algorithm delivers a simplistic empirical hand-based expression that can be used for future unseen data. The ANN and ANFIS algorithms are also used as a predictor to confirm the validity of the equation. A detailed and comprehensive database has been developed from peer-reviewed internationally published articles. This widespread database ensures the applicability of the model for new data. Statistical error checks are employed to verify the performance of the established models. In the end, a permutation feature analysis and a parametric study were also conducted to arrive at an accurate, reliable model.

### 1.2. Detailed Description of Machine Learning Algorithms (ANN, ANFIS, GEP)

**Figure 2:**
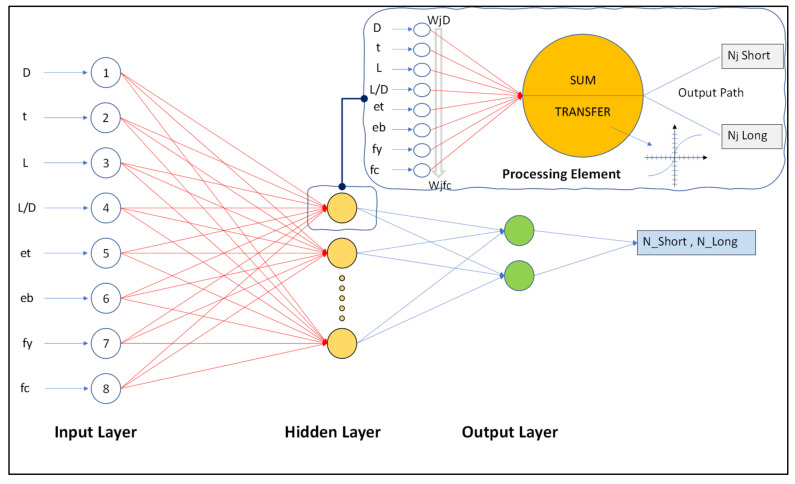
Schematic layout of feed-forward neural networks with eight explanatory variables.

Artificial Neural Networks (ANNs) are computer algorithms that anticipate and classify the issues concerned with the effective processing of data [42]. As its name indicates, ANNs are based on mathematical models based on the human brain’s neuron system [43]. ANN’s have various layers of processing elements or nodes. Figure 2 shows the three layers with arranged nodes, i.e., input layer/s, output layer/s and hidden layer/s. The input layers have independent variables, output layers’ target results, and hidden layers have concealed neurons/variables [44]. For each output (*N_st_/N_lg_*), eight inputs were selected, while in the hidden layer the input parameters (*D*, *t*, *L*, *L/D*, *e_t_*, *e_b_*, *f_y_* and *f_c_*) were multiplied by a suitable weight factor for connection. A threshold value at every node (*θ_j_*) is added to the weighted input values after their summation. The resultant input (*Ij*) is passed through the linear transfer function, which is called the transfer phase. The various activation transfer function (AFs) usually used in ANNs are the linear sigmoid, the stepped hyperbolic tangent and the logistic, among others [45]. An activation function is the key feature of a neural network which plays an important role in the artificial neural network model. It can be observed that these activated functions assist in appointing nonlinearity to the neural networks, due to which the selection of the appropriate activated function becomes very important [46]. Activated functions which have been used in the past include tangent hyperbolic and logistic sigmoid activated functions [47], the transcendental type parametric algebraic activated function [48], swish activated functions [49], and Multistate AF’s to improve the DNN models [50] etc.

In this research, the transfer functions used for the modelling of ANN models are TRANSIG and PURELIN. On the one hand, these transfer functions are capable of effectively increasing neurons in each layer and in each transfer function to improve the statistical indices of the training dataset. However, on the other hand, they also decrease the accuracy of the testing and validation datasets [51,52]. Dorofki et al. [53] observed that among various statistical functions, the performance of the Log-sigmoid transfer function was best, because these are differentiable, bounded and continuous. At the same time, Purelin TF gives much improved results. As a result, PE (*N_st j_* or *N_lg j_*) is obtained as the resulting output, and the input of a PE is basically the output of the previous PE. For the hidden and output layer, every neuron utilizes the Logistic function (Equation (1)) as an activated function [54]. Moreover, the complete process can be observed from Equations (1)–(3).

(1) $$f_{h}\left(z\right)=\frac{1}{1+e^{-z}}$$

(2) $$I_{j}=\left\{\left(w_{j\;D}*D+w_{j\;t}*t+w_{j\;L}*L\;\ldots \;w_{j\;fy}*f_{y}\right)\right\}+\theta_{j};\;\mathrm{Summation}$$

(3) $$N_{st\;j}\;or\;N_{lg\;j}=f\left(I_{j}\right);\;\mathrm{Transfer}$$

To achieve an output with the least minimum error, the best combination of weights is achieved by adjusting the weight to the set rules at the time when the information from the input layer is passed by the ANN in the training stage. Another training set is used to validate the trained model. The method and implementation of ANN modeling is discussed in greater depth elsewhere and is beyond the scope of this review [55,56,57].

An attractive computation intelligence modelling tool, adaptive neuro-fuzzy inference scheme (ANFIS), blends the learning capabilities of ANNs with the reasoning capability of fuzzy logic. ANFIS has a better prediction potential and is a better alternative for computing nonlinear complex problems with greater precision [58]. With similar learning capability as ANN, ANFIS learns from training data containing a multiplex model and then gives the solutions in a fuzzy interface system (FIS) [43]. In MATLAB R2020b there is a tool called ANFIS that can train the input and output entities for the best connection between both the parameters. A basic FIS consists of several stages. First are feeding inputs to aid in the fuzzification of fuzzy sets according to the activation of linguistic rules. Then basic laws are formulated by experts. These laws can also be derived from numerical results. Inference is the next step, which involves fuzzy mapping sets according to fixed laws. Finally, the fuzzy sets are defuzzied, which results in the final performance values.

**Figure 3:**
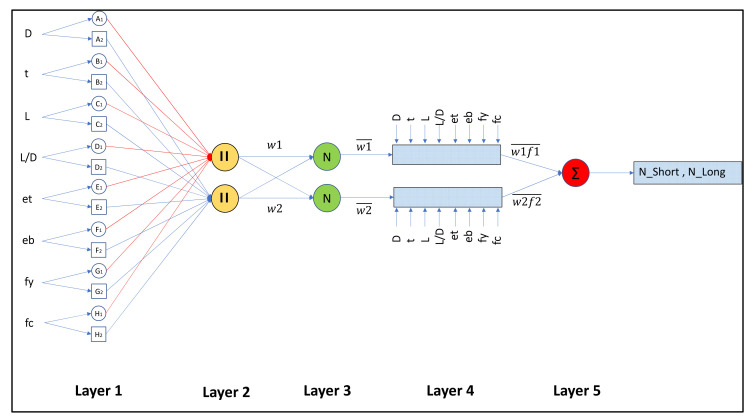
Sugeno ANFIS model schematic layout with eight explanatory variables.

The ANFIS technique is divided into five steps:

(a) data collection,
(b) ANFIS growth,
(c) variables selection,
(d) training and testing,
(e) results

to express values in another way. In addition, the schematic layout of the ANFIS model for eight input variables (*D*, *t*, *L*, *L/D*, *e_t_*, *e_b_*, *f_y_* and *f_c_*) is shown in Figure 3. The circle denotes the set nodes, while the square denotes the adaptive nodes. The two statements used for the presentation of the architecture of the ANFIS are IF-THEN statements are as follows.

Statement 1: IF (*D* is *A*_1_) and (*t* is *B*_1_) THEN,

(4) $$\left\{f_{1}=p_{1}\left(D\right)+q_{1}\left(t\right)+r_{1}\right\}$$

Statement 2: IF (*D* is *A*_2_) and (*t* is *B*_2_) THEN,

(5) $$\left\{f_{2}=p_{2}\left(D\right)+q_{2}\left(t\right)+r_{2}\right\}$$

where *f_n_* denotes the fuzzy outputs (*N_st_*, *N_lg_*) for the fuzzy inputs (*D*, *t*, *L*, *L/D*, *e_t_*, *e_b_*, *f_y_* and *f_c_*), according to the fuzzy statement, *A_i_* and *B_i_* denote the fuzzy sets, and *p_i_*, *q_i_*, and *r_i_* denote the arrangement elements determined in the training cycle.

An ANFIS model is made up of five layers [58], explained in detail below.

#### 1.2.1. Layer 1

This layer is also called the fuzzification layer. The adaptive Pes provide outputs in Equations (6) and (7), which describe the fuzzy membership functions of the input model parameters and the original fuzzy rule foundation.

(6) $$O_{k}^{1}={µ}_{Ak}\left(D\right),\;\;\;\;k=1,2$$

(7) $$O_{k}^{1}={µ}_{Bk-2}\left(t\right),\;\;\;k=3,4$$

where µ indicates the weight obtained by connecting the fuzzy membership function, and µ*_Ak_*(*D*) and µ_*Bk*-2_(*t*) differentiate the method of applying any fuzzy membership function. Equation (8) gives the µ*_Ak_*(*D*) for a bell-shaped membership function

(8) $${µ}_{Ak}\left(D\right)=\frac{1}{1+{\left\{\left(\frac{D-c_{k}}{a_{k}}\right)\right\}}^{b_{k}}}$$

where *a_k_*, *b_k_* and *c_k_* are the factors affecting this membership function.

#### 1.2.2. Layer 2

This layer’s output is the preset rules’ firing power for a given input pattern. The nodes in the second layer are constant and perform simple multiplication, with the output’s parameters mentioned below (Equation (9)),

(9) $$O_{k}^{2}=w_{k}={µ}_{Ak}\left(D\right).{µ}_{Bk}\left(t\right),\;k=1,2$$

#### 1.2.3. Layer 3

Following the pattern of the second layer, the third layer also has fixed nodes for normalizing the firing strength of the previous layer. Equation (10) represents the output:

(10) $$O_{k}^{3}={\bar{w}}_{k}=\frac{w_{k}}{w_{1}+w_{2}},\;k=1,2$$

#### 1.2.4. Layer 4

In this layer, considering the first order Sugeno model, nodes are adaptive. Their outputs are represented as products of normalized firing intensity and first-order polynomial, with the first order Sugeno model taken into consideration. As a result, the output is given by (Equation (11)):

(11) $$O_{k}^{3}={\bar{w}}_{k}f_{k}={\bar{w}}_{k}\left\{p_{k}\left(D\right)+q_{k}\left(t\right)+r_{i}\right\}$$

#### 1.2.5. Layer 5

In this layer, a fixed node summits the weighted magnitude of rules achieved from the prior layer, yielding Equation (12) as the model’s output.

(12) $$O^{5}=\overset{2}{\underset{k=1}{\sum }}{\bar{w}}_{k}f_{k}=\frac{\sum_{k=1}^{2}w_{k}f_{k}}{w_{1}+w_{2}}$$

It is worth noting that only the first and fourth layers of the ANFIS architecture are adaptive. In the first layer, the three adaptable parameters *a_k_*, *b_k_*, and *c_k_*, also known as premise parameters, are linked to input membership functions. Similarly, the three adaptable parameters *p_k_*, *q_k_*, and *r_k_*, also known as consequent parameters, are analogous to first-order polynomials and are found in the fourth sheet [59].

The gene expression programming approach, which is founded on Darwin’s evolution theory and Mendel’s genetic theory, is the most intellectually appealing computational knowledge algorithm [60,61] There are two languages in GEP: (a) the gene’s language, and (b) the expression tree’s (Ets) language, and understanding one requires knowledge of the other’s sequence or structure [62]. The following are the fundamental steps involved in traditional GEP modelling. A typical gene or chromosome contains two parts i-e:

(a) Head consisting of function or terminal symbols
(b) Tail containing only the terminal symbols.

The complexity of each parameter is represented by head size and the number of genes that control the number of sub-Ets.

**Figure 4:**
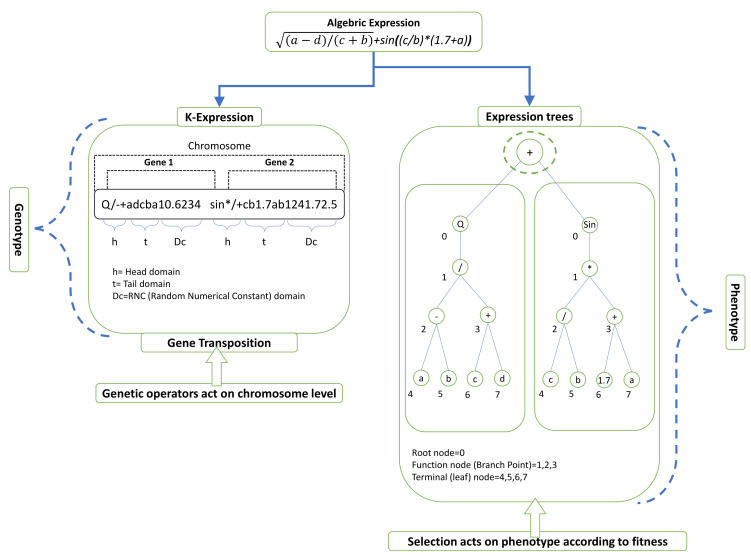
Illustration of a mathematical equation and its equivalent expression tree (ET).

Figure 4 shows how the chromosomes have set lengths that can be easily converted into an algebraic expression [63]. Every GEP gene has a series of words that are adapted from the function set; for example, arithmetic operations (+, −, ×, ÷), Boolean logic functions (AND, OR, NOT, etc.), trigonometric functions (cos, sin, ln), conditional functions (IF, THEN, ELSE), etc. [64].

The chromosomes are then expressed by Ets that come in a range of shapes and sizes. Then, in line with their percentages, the principal genetic operators of crossover, mutation, transposition, and recombination (1-point, 2-point, and gene recombination) are performed on the chromosomes [65]. Figure 4 illustrates a common expression tree (ET) and describes the crossover and mutation processes. Equation (13) also shows how the ET is expressed using Karva notation or a K-expression [36].

(13) $$ET_{GEP}=log(i-\frac{3}{j})$$

When the stopping condition (the maximum number of generations or a satisfactory solution) is reached, the whole process is finished [66]. If the termination conditions for achieving the optimum iteration or the favorite fitness value are not satisfied, then the Roulette wheel procedure is used, which chooses the viable chromosomes of the first generation and moves them on to the next generation [67]. This method will be repeated for a certain number of generations or before the right solution is found [68].

### 1.3. The Aim of the Research

It follows from the above review that the variety of existing models does not allow rational predicting of the ultimate axial capacity of uniaxially loaded CFST columns. The article aimed to develop a prediction Multiphysics model for the circular CFST column by using Artificial Neural Network (ANN), Adaptive Neuro-Fuzzy Inference System (ANFIS) and Gene Expression Program (GEP).

## 2. Methods

### 2.1. Description and Division of Collected Data

A robust database is needed to successfully apply the machine learning algorithms (ANN, ANFIS, GEP). Thai et al. [6] collected the most recent experimental data of CFST columns and combined it with the existing database [69]. The Thai et al. [6] database is comprised of more than 3100 tests performed on CFST columns of different classes. A total of 1667 experimental results were extracted from the existing database, which comprised two different classes: circular short (702) and long (965) CFST columns loaded concentrically and eccentrically. The geometric features include physical dimensions such as length (*L*), tube thickness (*t*) and tube diameter (*D*), and eccentricities at end supports (*e_t_*, *e_b_*). The material properties of steel and concrete include the yield stress (*f_y_*) and compressive strength (*f_c_*) of concrete. The concrete compressive strength obtained from the experimental tests collected from the literature was based on both available cylinder and cube specimens. Cube strength was converted to cylinder strength through related conversion factors. Furthermore, cylinder strength was used in the design equations [70] to avoid errors. Other material properties, such as steel and concrete moduli and steel ultimate stress, were considered of minor significance. In the case of concrete, the compressive strength for all the specimens is given, and the modulus is directly affected by this compressive strength, so there is no need to establish any relation between compressive strength and modulus of concrete, and only strength was incorporated in the model as a significant factor. A similar strategy was used to eliminate the need for steel’s ultimate stress, while in the case of steel modulus, for example, 200 Gpa are probable, and this value is normal for all steel grades used in all columns.

For each class, descriptive statistics like distribution shape (kurtosis and skewness), central tendency (mean and median), dispersion of data (Standard deviations) and data extremities (maximum and minimum) for different geometric and material characteristics are provided in Table 1. For instance, the diameter ranges from 44.5 mm to 1020 mm for both classes, and the thickness ranges from 0.5 mm to 16.5 mm and 0.5 mm to 13.3 mm for long and short CFST columns, respectively. Similarly, the compressive strength range in filled concrete for both classes is from 7.7 MPa to 193.3 MPa, while that for yield strength of steel tube for long and short CFST columns is from 178.3 MPa to 853 MPa and from 185.7 MPa to 853 MPa, respectively. The range of *L/D* ratio is witness to the difference between short and long CFST columns. The reader is advised to note that these ranges surpass the design codes currently in use. As a result, this database can be used to build an ANN, ANFIS and GEP model with enhanced prediction capabilities that are more inclusive than codal provisions. It should be remembered that the magnitude eccentricity is a part of the affecting parameters in both classes. Therefore, the developed models can be confidently used for both axial and moment capacity of circular CFST columns. Skewness and kurtosis are related to the distribution of data. If the larger portion of the data for a particular variable is to the left of the mean, then this shows positive skewness (right tailed). Furthermore, if most of the data is to the right of mean value, then this shows negative skew (left tailed), and skewness is zero for perfectly symmetrical distribution (normal distribution). At the same time, the kurtosis indicates the heaviness of the tail related to the normal distribution. The leptokurtic or positive kurtosis dictates that the data is higher than the normal distribution, whereas the platykurtic or negative kurtosis reveals that the data is flatter than the normal distribution. The values of kurtosis and skewness for each input and output is also provided in Table 1. After constructing a reliable database, the available datapoints are divided into two sets, i.e., training and testing set [71].

### 2.2. Structure of ANN, ANFIS and GEP Models

The specification of the significantly affecting input parameters is the first step in designing the appropriate model. The *N_st_* and *N_lg_* were found to be dependent on the following factors (Equation (14)):

(14) $$N_{\mathrm{st}}\;\mathrm{or}\;N_{\mathrm{lg}}\;(\mathrm{kN})=f(D,\;t,\;L,\frac{L}{D}{,\;e}_{t}{,\;e}_{b}{,\;f}_{y}{,\mathrm{and}\;f}_{c})$$

Here, *D* is the diameter of the tube, *t* is the thickness of the tube, *L* is the length of the tube, *L/D* is the ratio between length and diameter of the tube, *e_t_* and *e_b_* is eccentricity at top and bottom face, *f_y_* is the yield strength of tube, and *f_c_* is the compressive strength of the tube.

Both ANN and ANFIS simulations were performed in the MATLAB R2020b environment using the neural network and fuzzy logic toolbox, respectively. The 702 experimental records of short CFST columns were randomly divided into 70% training (495 datapoints) and 30% testing (207 datapoints), and similarly for second class, i.e., long CFST columns, out of a total of 965 experimental records, 70% (676 datapoints) and 30% (289 datapoints) were accumulated in the training and testing set, respectively [36]. The training accuracy and time taken to train the model are essential [72] for comparison of the performance of each model. The input layer in this analysis had eight input nodes, one for each of the model inputs (*D*, *t*, *L*, *L/D*, *e_t_*, *e_b_*, *f_y_*, *f_c_*), and the output layer had *N_st_* and *N_lg_* for ANN. After using the Levenberg-Marquardt algorithm and choosing random data division, the number of hidden neurons was set to ten. In addition, the network form was chosen as feed-forward back-propagation. To achieve a better output at the required number of hidden layers, trial and error methods should be used [54]. Table 2 lists the statistical parameters of modelling for ANNs in this research.

ANFIS provides only a single output; unlike ANNs, the outputs were handled separately using the same set of input parameters in both ANN and ANFIS models. The sub-cluster FIS was first generated because the database contained a huge number of data points, in which subtractive clustering with a hybrid optimization technique (least square and back-propagation technique) was used to train the FIS by building a triangular membership function (trimf) [73]. Furthermore, Venkatesh and Bind [42] also recommend using the grid portioning approach in which the maximum number of input parameter is taken as six. The various setting parameters for the training phase are presented in Table 2**.**

GeneXproTools version 5.0 was used to implement the GEP algorithm [74,75]. From data loading to code generation, GeneXproTools is a versatile data processor that supports categorical variables and missing values, greatly enhancing the performance and accuracy of the modelling process [76]. It can generate multiple models from large heterogeneous data and locate code in programming languages such as MATLAB, C++, and Visual Basic [54]. The parameters in the GEP algorithm were finalized based on previous literature guidelines and several initial runs [41,77]. The initial optimum combination of GEP parameters was calculated using the trial-and-error method. The effect of a single GEP parameter on prediction accuracy was then investigated using the optimum combination of parameters. Finally, the proposed model was formulated using the finalized optimum combination of GEP parameters for obtaining basic mathematical expressions to predict the bearing capacity of CFST columns. The model’s complexity increases as the number of chromosomes, head size, and genes increase, and hence the length of the running program is determined.

Furthermore, multiple evolved models are strongly influenced by head size and genes. The error in the formulated model is reduced by the higher values of these setting parameters, which results in a higher value of the coefficient of regression. Table 2 shows the unique values for the parameters in the GEP algorithm for both classes of CFST columns considered in this study.

### 2.3. Evaluation of Models through Statistical Measures

Statistical performance for the *N_st_* and *N_lg_* prediction (ANN, ANFIS and GEP models) were measured using five standard statistical metrics, including correlation coefficient (*R*), determination coefficient (*R*^2^), root mean square error (RMSE), mean absolute error (MAE), relative squared error (RSE), mean absolute percent error (MAPE) and relative root mean square error (RRMSE) in the training testing sets [77,78,79]. In addition, for all the proposed models, a performance index (*PI*) has been calculated as another metric, ruled primarily by RRMSE and *R* [36]. Equation (15) to Equation (21) define these performance measures:

(15) $$\mathrm{MAE}=\frac{\sum_{i=1}^{n}\left|p_{i}-q_{i}\right|}{n}$$

(16) $$\mathrm{RMSE}=\sqrt{\frac{\sum_{i=1}^{n}{\left(p_{i}-q_{i}\right)}^{2}}{n}}$$

(17) $$\mathrm{RSE}=\frac{\sum_{i=1}^{n}{\left(p_{i}-q_{i}\right)}^{2}}{\sum_{i=1}^{n}{\left({\bar{p}}_{i}-p_{i}\right)}^{2}}$$

(18) $$\mathrm{RRMSE}=\frac{1}{\left|\bar{e}\right|}\sqrt{\frac{\sum_{i=1}^{n}{\left(p_{i}-q_{i}\right)}^{2}}{n}}$$

(19) $$\mathrm{MAPE}=\frac{1}{n}\overset{n}{\underset{i=1}{\sum }}\left|\frac{q_{i}-p_{i}}{q_{i}}\right|\times 100$$

(20) $$R=\frac{\sum_{i=1}^{n}\left(p_{i}-{\bar{p}}_{i}\right)\left(q_{i}-{\bar{q}}_{i}\right)}{\sqrt{\sum_{i=1}^{n}{\left(p_{i}-{\bar{p}}_{i}\right)}^{2}\sum_{i=1}^{n}{\left(q_{i}-{\bar{q}}_{i}\right)}^{2}}}$$

(21) $$\mathrm{PI}=\frac{\mathrm{RRMSE}}{1+R}$$

Here, $p_{i}$ and $q_{i}$ are the *i*th predicted and expected outcomes, respectively, ${\bar{p}}_{i}$ and ${\bar{q}}_{i}$ are the average of predicted and expected outcomes, and *n* is the total number of experiments. To determine the relative correlation between models and experimental outputs ($p_{i}$ and $q_{i}$), the performance of *R* is used. When *R* > 0.8, the predicted and expected values are highly correlated [80]. However, *R* is insensitive to the division and multiplication of outcomes [77]. Thus, *R*^2^ was used because of its impartial assessment and comparatively better performance. *R*^2^ values equal to 1 and closer to each other demonstrate that much of the model’s variation between input parameters was used [81]. In addition, RMSE is a common metric since significant errors in comparison to smaller errors are resolved very effectively. RMSE closer to 0 indicates that the prediction error is negligible [58]. It does not, however, ensure optimum efficiency in any conditions. MAE was also calculated and is enormously advantageous in the presence of smooth and continuous data [82]. To sum up, a greater value of *R* and smaller values for RMSE, MAE, RSE, and RRMSE provides a better standard calibration for model performance. In addition, Gandomi et al. [83] proposed that *PI* ranges from 0 to infinity and closer to zero indicate a good model performance.

In a range of machine learning techniques, due to unnecessary data training, the models appear to overfit [84] and lead to lower training error values and greater testing error values. In order to choose the best predictive model that can solve overfitting, a minimized objective function (*OF*) is used, as shown in Equation (22) [36,85]:

(22) $$OF=\left(\frac{n_{T}-n_{v}}{n}\right){\mathrm{PI}}_{T}+2\left(\frac{n_{v}}{n}\right){\mathrm{PI}}_{v}$$

Here *T* and *V* (subscripts) correspond to training and testing set points, and n represents the total number of records. The better-predicted formula must have a lower OBF value (nearly equals to 0), as the consequence of *R*, RRMSE and proportional percentage of dataset records are considered. Eight different suitable parameter combinations were executed, and the least *OF* was chosen in this study.

## 3. Results and Discussion

### 3.1. Regression Analysis of ANN, ANFIS and GEP Model

**Figure 5:**
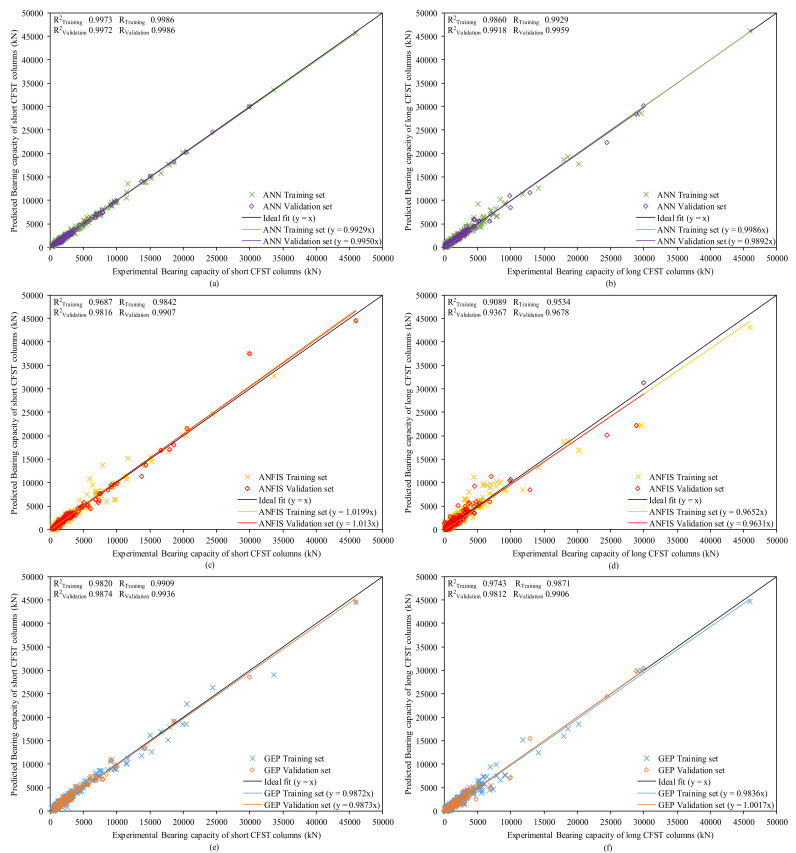
Regression plot between actual and predicted bearing capacity of (**a**) ANN model for short CFST columns (**b**) ANN model for long CFST columns (**c**) ANFIS model for short CFST columns (**d**) ANFIS model for long CFST columns (**e**) GEP model for short CFST columns and (**f**) GEP model for long CFST columns.

The regression plots between actual and predicted bearing capacities for short and long CFST columns of ANN, ANFIS and GEP models are clearly presented in Figure 5. The equation for the slope of the regression lines between actual and predicted bearing capacity for both training data and testing data has been shown in all plots. The closeness of the datapoints near the regression line drawn at 45° indicates the better performance of the established models [58]. The slope should be closer to 1 for an ideal fit [77,86] and for a strong correlation. For all three proposed models, a strong correlation can be seen as depicted from the slope of the regression lines. The values of slope for both stages, i.e., training and testing set, are quite similar in all the proposed models showing that the models are efficiently trained and hold a high generalization capacity. The spread of the datapoints in the training and testing set also shows that the issue of overfitting has been diminished.

Moreover, the coefficient of determination (*R*^2^) for all the proposed models is greater than 90% in both training and testing stages following the trend: *R*^2^(ANN) > *R*^2^(GEP) > *R*^2^(ANFIS), reflecting the shortcoming of neural and fuzzy arrangement in the projected ANFIS model. The mean correlation coefficient (*R*) in the projected models for *N_st_* is also maximum for ANN (0.9986) tracked by GEP (0.9922) and ANFIS (0.9874). For a stronger correlation, the *R*-value will be higher (i.e., *R* > 0.8) for an acceptable model [77,87,88]. In the case of *N_st_*, the *R*^2^ for ANN model is highest, i.e., 99.73% and 99.72% for training and testing data, respectively, and 98.20% and 98.74% for the GEP model, respectively.

### 3.2. GEP Based Formulation of Bearing Capacity of CFST Columns

**Figure 6:**
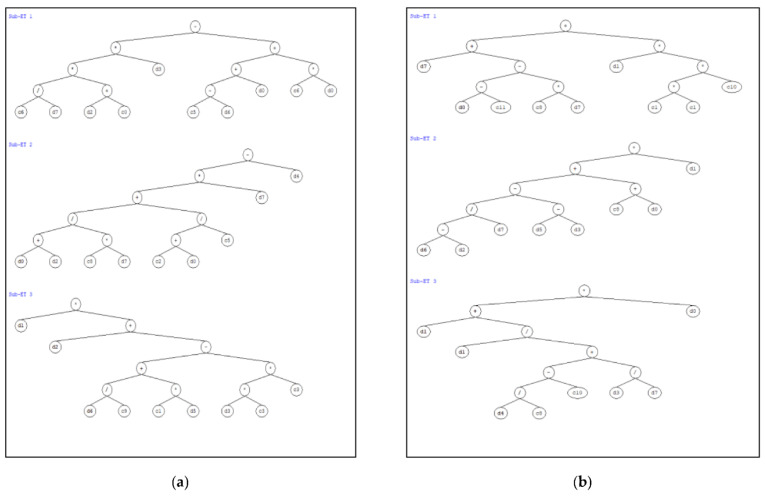
Representation of expression trees (**a**) Short circular CFST (**b**) Long circular CFST.

The two GEP based empirical formulae for future prediction of bearing capacity of short and long circular CFST columns are derived using the GEP algorithm. The sub-ETs’ links to these formulae for *N_st_* and *N_lg_* use four basic mathematical operations, i.e., +, −, × and ÷, as presented in Figure 6. The ETs in Figure 6a,b corresponding to three different numbers of genes with addition used as a linking function, are decoded to derive a respective mathematical equation for *N_st_* and *N_lg_* as shown in Equations (23) and (24). Based on the total number of records, the projected formulae are in close agreement with standard limits for an ideal model and can be confidently and reliably used for the prediction of bearing capacity of short and long circular CFST columns [64,89,90].

(23) $$N_{st}\left(kN\right)=\left(\left(\frac{-18.9L+11525.98}{f_{c}}\times \frac{L}{D}\right)-1711.5+f_{y}-D+\left(18.94\times D\right)\right)\;+\left(f_{c}\times \left(\frac{D+L}{-0.38\times f_{c}}+\frac{-45.67+D}{5.42}\right)-e_{t}\right)\;+\left(t\times \left(L+\frac{f_{y}}{5.48}-\left(3.07\times e_{b}\right)-\left(\frac{L}{D}\times 88.45\right)\right)\right)$$

(24) $$N_{lg}=\left(\left(f_{c}+D+9.55+\left(7.1\times f_{c}\right)\right)+\left(t\times 225.8\right)\right)+\left(\left(\frac{f_{y}-L}{f_{c}}-e_{b}-\frac{L}{D}-11.82+D\right)\times t\right)\;+\left(\left(t+\frac{t}{\frac{e_{t}}{4.03}+0.54+\frac{L/D}{f_{c}}}\right)\times D\right)$$

### 3.3. Performance Evaluation of Proposed Models Using Statistical Indicators

The reliability and accurateness of the models greatly depend on the number of data points [77]. In this research, a maximum number of records, i.e., for short CFST columns 495 training and 207 testing records and for long CFST columns 676 Training and 289 testing records, are used for the development of models and, hence, better accuracy has been achieved. It is suggested in the literature that, for a reliable model, the ratio between several records and input variables in both training and testing should be at least 5 [91]. In the training and testing stage of this study, the specified ratio is far beyond the limit, i.e., equal to 61.88 and 25.88, respectively, for *N_st_* and 84.5 and 36.13, respectively, for *N_lg_*,

The studies suggested that *R* or *R*^2^, enumerates the linear dependency of response and explanatory variables. An acceptable value of *R* greater than 0.8 shows a strong correlation between actual and predicted values [92]. Thus, the evaluation of the proposed models based on the slope of the regression line and regression or correlation coefficient is insufficient [92]. Therefore, the developed models are also assessed using different statistical metrics for evaluating their robustness.

#### 3.3.1. ANN Model

**Figure 7:**
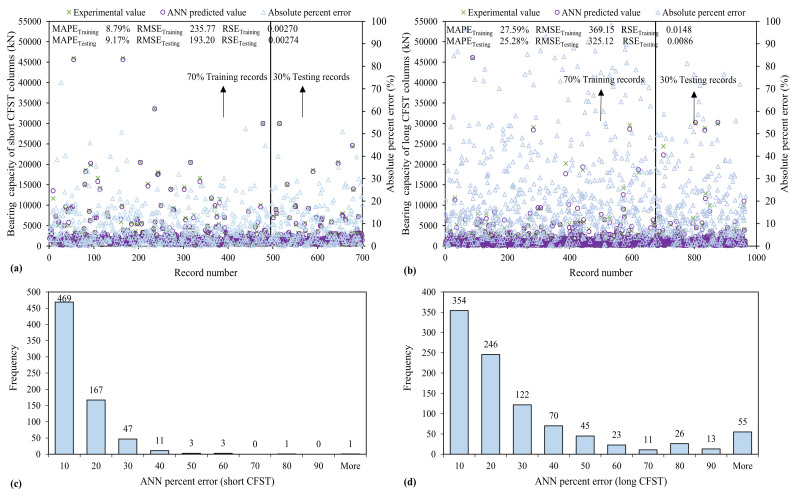
Variation of mean absolute error and error histograms of bearing capacity established using ANN algorithm (**a**,**c**) short CFST (**b**,**d**) long CFST columns.

It can be seen from Figure 7, that for short CFST columns, the R exceeds 0.90 and is nearly equal to 1, which shows that the ANN model has a better prediction capability for training and testing data sets that is perfectly equal to 0.9986 for training and testing both sets, which witnessed the outburst performance of the ANN model. Similarly, in the case of long CFST columns, these are 0.9929 and 0.9959 for the respective datasets. To interpret the statistics of MAPE, the absolute percent error plot and the error histogram of percent error are shown in Figure 7c,d. The percent error histograms indicate 58% and 36% ANN predicted values for *N_st_* and *N_lg_*, respectively, have an error less than 10% As reflected in Figure 7a,b, the error values are scattered near zero, showing the outburst performance of the developed multi-physics-based ANN model. Furthermore, both the MAE and RMSE in Table 3, enumerate the magnitude of average error values and have their own importance. The RMSE squared the error before average and gave more weightage to larger error values [93]. At the same time, MAE gives low weightage to larger error values and is always lower than RMSE [80,93]. For the ANN model for *N_st_*, *_testing_MAE_testing_* (145.96) is lower than *RMSE_testing_* (193.20), satisfying the stated condition. Similarly, these values for *N_lg_* are (196.22) and (325.12), respectively. The *RSE_testing_* of ANN models for *N_st_* and *N_lg_* is also minimal and nearly equals zero, i.e., (0.00274) and (0.0086), respectively. The details of the statistical analysis of all the proposed models for *N_st_* and *N_lg_* in both stages (training and testing), are provided in Table 3.

#### 3.3.2. ANFIS

**Figure 8:**
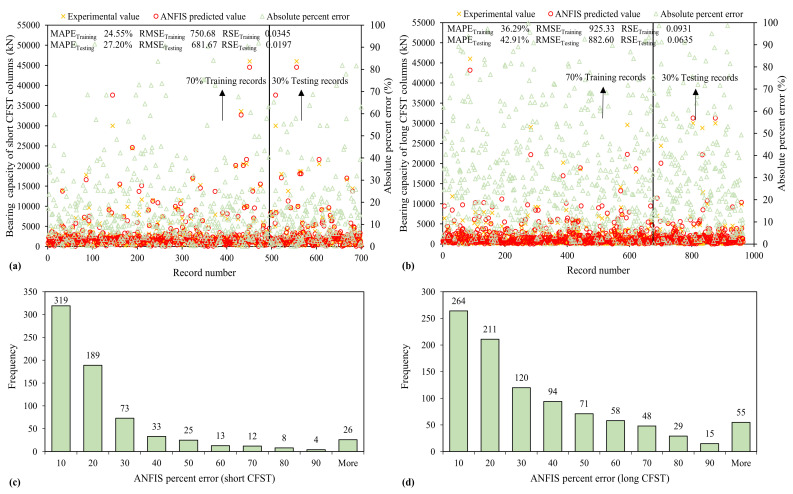
Variation of mean absolute error and error histograms of bearing capacity established using ANFIS algorithm (**a**,**c**) short CFST (**b**,**d**) long CFST columns.

The ANFIS model also delivers a good result based on performance criteria. Along with high values of *R* exceeding 0.90, lower error statistics were recorded. Unlike ANN and GEP, the magnitude of errors (MAE, RMSE and RSE) is higher. The percent error histogram and distribution of MAPE is shown in Figure 8, indicating that 46% and 27.3% ANFIS predicted values for *N_st_* and *N_lg_*, respectively, have percent error less than 10%.

In comparison with ANN models, it gives more high-error values. However, as presented in Figure 8, the absolute percent error runs near the axis showing that the overall performance is satisfactory. As stated in Table 3, the *MAE_testing_* and *RMSE_testing_* of ANFIS models is (55.6)% and (71.7)% greater than that of ANN models in the case of *N_st_* and (60.8)% and (63.2)% greater in the case of *N_lg_*, respectively. Like the ANN model, the *RSE_testing_* for ANFIS models is also near to 0. Thus, based on the above facts, the prediction of bearing capacity of short and long CFST columns can also be obtained through these reliable and accurate models.

#### 3.3.3. GEP Model

**Figure 9:**
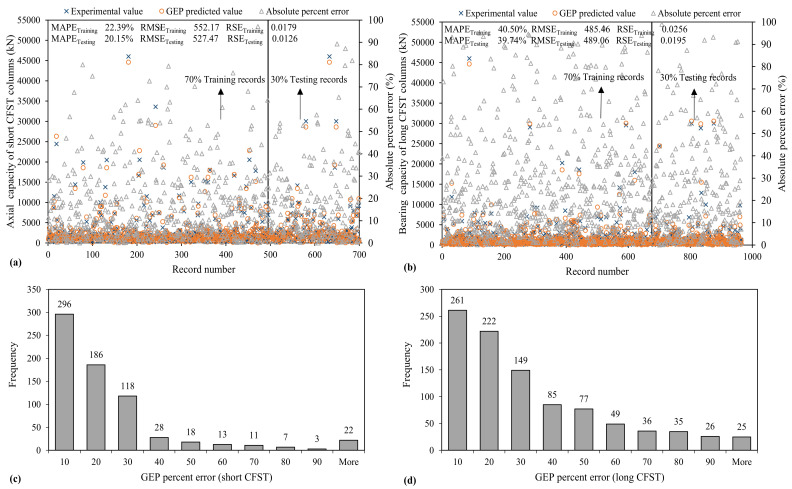
Variation of mean absolute error and error histograms of bearing capacity established using GEP algorithm (**a**,**c**) short CFST (**b**,**d**) long CFST columns.

The GEP model provides better results than the ANFIS model but is worse than the ANN model based on *R* or *R*^2^ and the magnitude of error statistics. However, as shown in Figure 9, around 42% and 27% (nearly equal to the results deduced for the ANFIS model) of the GEP predicted values for *N_st_* and *N_lg_* have percent error below 10%, which are less than the ANFIS model. In the GEP model, the RMSE and RSE values for the testing set are lower than the ANFIS model by 22.6% and 36% for *N_st_*, respectively, and 44.6% and 69.3% lower for *N_lg_*, respectively.

### 3.4. Comparison of Models Using External Testing Criteria

The literature recommended an RRMSE of between 0 and 0.1 for an excellent model, and for a good model between 0.1 and 0.2. As shown in Table 3, following RRMSE, the performance of the three proposed models for both classes can be categorized as ANN followed by GEP and ANFIS. Gandomi and Roke [36] also classified the machine learning models as Good, if the *PI* and *OF* are less than 0.2 (*OF* nearly equal to 0 denotes an ideal model). The *PI* and *OF* values of GEP models for the *N_st_* and *N_lg_* fall within the prescribed limit and show an outstanding performance. However, the *PI* in the training and testing stage of the ANFIS model for *N_lg_* are equal to 0.2990 and 0.2652. Its *OF* value is also equal to 0.2700. In the ANN model for *N_st_*, the *PI* in the testing stage is 0.361, which is considerably higher than the prescribed limit. Consequently, the *OF* for the model is 0.2300. Thus, the performance of the ANN and ANFIS model based on *PI* and *OF* is ambiguous and marked as satisfactory, although the ANFIS model for short and ANN model for long CFST columns perform well. Lewis [94] suggested that the model can be categorized as either excellent prediction $\left(\mathrm{MAPE}\leq 10\%\right)$, good prediction (10%<MAPE≤20%), acceptable prediction (20%<MAPE≤50%) and inaccurate prediction (50%<MAPE) [79,95]. In accordance with the mentioned criteria of model categorization based on MAPE, the ANN provides excellent forecasting results for *N_st_*, while all other models including *N_lg_*-ANN falls in the “acceptable prediction” category.

The GEP suggested models for forecasting bearing capacity surpasses the other two models since this method provides simplified expressions, presented as Equations (23) and (24). With these formulas, the overall time needed for both the *N_st_* and *N_lg_* tests utilizing the corresponding GEP models is significantly quicker considering the time needed by the traditional test technique [41]. Thus, the suggested mathematical formulas provide a feasible fast method for finding *N_st_* and *N_lg_*.

Furthermore, several other external testing criteria for the GEP model were suggested in the literature and are presented in Table 4. Mollahasani, Alavi [96] stated that at least one of the regression slopes lines (*k’/k*) moving through the origin must reach one. The results of performance indicators (*m* and *n*) must not be above 0.1. The criterion of third external testing presented in this research was given by [97], which implies an *Rm* > 0.5 and in this research, this is fulfilled by both GEP models. Likewise, the square correlation coefficient amongst predicted and actual data (*R*_0_^2^), and amongst experimental and actual data (*R*_0_′^2^) must be nearer to 1 [76]. It can be observed in Table 4 that the suggested GEP models satisfy all the necessary criteria, thus indicating the better level of accurateness of both the models.

### 3.5. Sensitivity and Parametric Study of GEP Models

Sensitivity analysis shows how an output of a proposed model is sensitive to a certain change in its input parameters. It ranks parameters by capturing the behavior of the model in response to the changes in a particular parameter and indicates the effectiveness of each parameter [54,72,98]. The comparative impact of the input variables included in this research on the short and long circular CFST columns is evaluated by executing sensitivity analysis (*SA*) on the GEP models utilizing Equations (24) and (25):

(25) $$N_{i}=f_{max}\left(y_{i}\right)-f_{min}\left(y_{i}\right),$$

(26) $$SA\;\left(\%\right)=\frac{N_{i}}{\sum_{n}^{j=1}N_{j}}*100.$$

**Figure 10:**
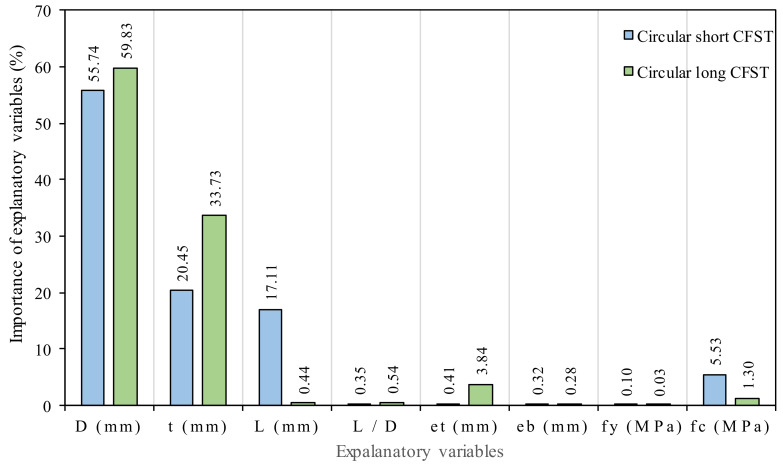
Relative importance of input variables on the bearing capacity of short and long circular CFST columns.

Here, $f_{max}\left(y_{i}\right)$ is the maximum Model projected output for the ith input parameter and $f_{min}\left(y_{i}\right)$ is the minimum model projected output for the ith input parameter, while the rest of the input parameters are kept as 1. The relative importance of input variables on the bearing capacity of short and long circular CFST columns is graphically displayed in Figure 10, which shows that the diameter, thickness, and length are the most sensitive parameters in short columns with a value of relative contribution of 55.7%, 20.45% and 17.1%, respectively, while in the case of slender columns, only diameter and thickness are sensitive parameters with a value of relative contribution of 59.8% and 33.7%, respectively. The sensitivity analysis also reveals that the influence of strength of steel (*f_y_*), and eccentricity at bottom (*e_b_*) parameters is negligible for both models with relative contribution of 0.09%, 0.03%, 0.3% for short and 0.03%, 0.3%, 0.5% for long CFST columns, respectively. The impact of length (*L*) in long columns and eccentricity at the top (*e_t_*) in short columns is also less, with a relative contribution of 0.44% and 0.41%, respectively. According to [99], the bearing capacity of CFST columns is mainly governed by the diameter and thickness of the steel tube.

**Figure 11:**
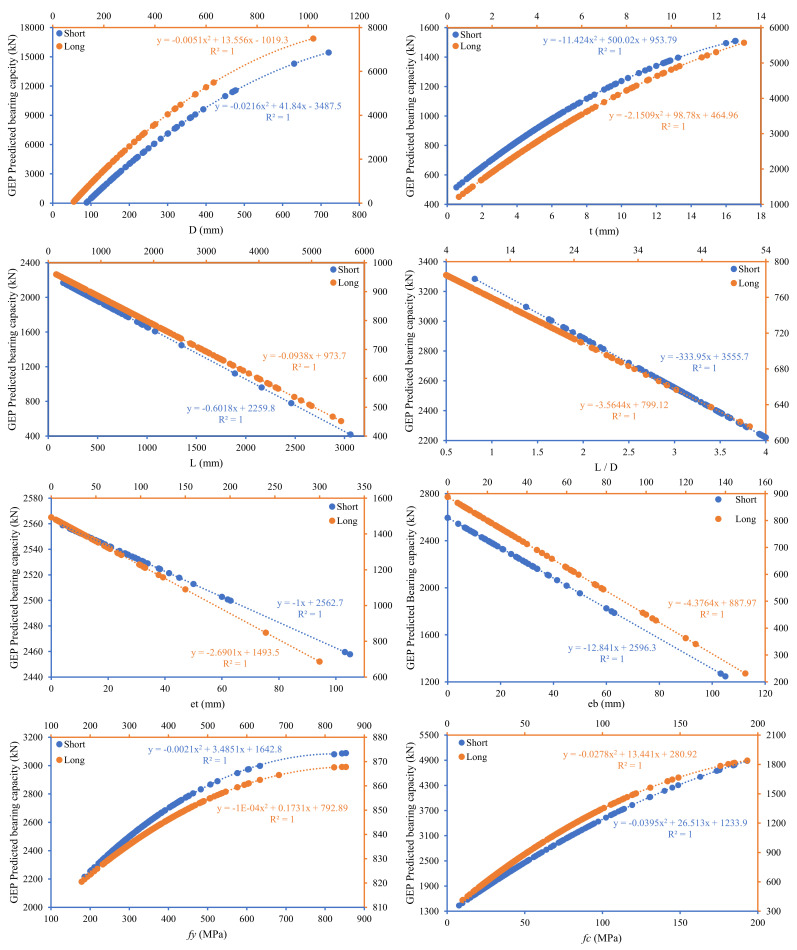
Summarized parametric study for formulation of bearing capacity of short and long circular CFST columns using Gene expression programming (GEP) in reference to the input variables (*D*: diameter of tube, *t*: thickness of tube, *L*: length of tube, *L/D*: length to diameter ratio, *e_t_* and *e_b_*: eccentricity at top and bottom surface, *f_y_*: yield strength of tube, *f_c_*: compressive strength of concrete).

Secondly, parametric analysis is performed to verify the efficiency of input variables and the strength of the GEP model. This is accomplished by changing one parameter with a particular increment while keeping all other parameters constant at their mean values. The main characteristics of the input parameters of CFST columns are material and geometric properties. Figure 11 indicates the prediction ability of GEP models for simulation of *N_st_* and *N_lg_* with varying input variables, i.e., *D*, *t*, *L*, *L/D*, *e_t_*, *e_b_*, *f_y_*, and *f_c_*. It is well understood that *D* and *t* are important factors controlling the bearing capacity of CFST columns. The capacity of both short and long columns follows a degree polynomial curve with variation diameter and thickness of the tube. Similar trends were also noticed for the compressive strength and yield strength of the steel tube. Increasing the compressive strength of concrete will divert the failure control mode to the yield strength of the steel tube and vice versa. The strength of the concrete inside the CFST is responsible for the stiffness of the CFST columns [100]. Stiffness rises along with concrete compressive strength, yet columns fracture owing to concrete crushing and brittle behavior when filled with high strength concrete. However, regardless of the length to diameter ratio, an increase in concrete core strength enhances the strength of filled columns to a greater extent. Linear decreasing pattern was observed for variation in the length or length to diameter ratio of the steel tubes with the prescribed limits. The effect of the eccentricity at the top or bottom face of the column also adversely affecting the capacity of CFST columns. Compared to zero eccentricity, in the case of eccentric loading the contact stresses will not be distributed non-uniformly, causing outward buckling [101]. Moreover, changes in the diameter and thickness of the steel tube greatly influence the bearing capacity of CFST columns for *N_lg_* and *N_st_*, as observed and stated by many researchers in the past [6].

Hence the results of the current study are similar to those found in past research studies accumulated in the database [6]. The parametric analysis also effectively captures the input parameters for the prediction of *N_lg_* and *N_st_*.

## 4. Conclusions

In this study, prediction models for the bearing capacity of circular CFST short (*N_st_*) and long columns (*N_lg_*) were developed through ANN and ANFIS and GEP. Two databases were extracted from the literature by collecting 702 datasets of short and 965 datapoints of long circular CFST columns. The conclusion is drawn below.

1. The GEP model can efficiently predict *N_st_* and *N_lg_* with high accuracy and best performance. Moreover, the bearing capacity prediction model from GEP is better than the ANFIS and ANN models. The diversity of the GEP technique can be seen from the simplified formulation, with higher accuracy and correlation among the experimental and predicted data with the consideration of linear and non-linear data.
2. The statistical indicators used to evaluate the performance of the model were mean absolute error (MAE), root square error (RSE), root means square error (RMSE), correlation coefficient (*R*), relative root mean square error (RRMSE), performance index (*PI*) and objective function (*OF*). The *PI* of the predicted *N_st_* by GEP, ANN and ANFIS for training are 0.0416, 0.1423, and 0.1016, respectively, and for N_lg_ these values are 0.1169, 0.2990 and 0.1542, respectively. Corresponding *OF* values are 0.2300, 0.1200, and 0.090 for *N_st_*, and 0.1000, 0.2700, and 0.1500 for *N_lg_*. The superiority of the GEP method to the other techniques can be seen from the fact that the GEP technique provides suitable connections based on the practical experimental work and does not undertake prior solutions. In reference to MAPE indicator, the ANN provides excellent forecasting results for *N_st_*, while all other models including *N_lg_*-ANN fall in the “acceptable prediction” category.
3. Sensitivity analysis was performed and the following input importance with increasing pattern was observed for *N_st_*: *D* (55.45) > *T* (20.45) > *L* (17.109) > *f_c_* (5.526) > *e_t_* (0.41) > *L/D* (0.34) > *e_b_* (0.32) > *f_y_* (0.096); whereas, in the case of *N_lg_*, it followed the order: *D* (59.83) > *T* (33.73) > *e_t_* (3.844) > *f_c_* (1.302) > *L/D* (0.541) > *L* (0.443) > *e_b_* (0.282) > *f_y_* (0.033). Parametric analysis showed a trend similar to the findings in previous literature. The effect of input parameters on the bearing capacity of circular short (*N_st_*) and long (*N_lg_*) CFST columns was studied. Thus, it can be concluded from this research that artificial intelligence techniques can be effectively employed to solve various complex engineering problems, especially in structural and material engineering. A simple, reliable, and accurate model can be developed which can perform better on unseen data.
4. The overall comparison shows that the most reliable and accurate technique for developing prediction models is GEP. The prediction models developed through the GEP technique are simpler than ANN and ANFIS models. It is, therefore, suggested that the developed GEP equations (Equations (22) and (23)) are used in routine design for circular short (*N_st_*) and long (*N_lg_*) CFST columns with eccentric loading using simple geometric and material properties. These models can replace tedious, time consuming and costly experimental work for finding the bearing capacity of CFST columns.

It is recommended to study tother AI techniques such as Random Forest Regression (RFR) and Multi-Expression Programming (MEP), etc., on the same data, and the models should be compared for accuracy, reliability, and ability to correlate the predicted data with the experimental data.

## Figures and Tables

**Table 1:** Descriptive statistical analysis of explanatory variables and response.

Category	Parameters	Mean	Median	Max	Min	S.D.	Kurtosis	Skewness
**Long**	**Inputs**							
	*D* (*mm*)	147.2	121.0	1020.0	44.5	89.9	31.78	4.38
	*t* (*mm*)	4.4	4.0	16.5	0.5	2.4	5.12	1.79
	*L* (*mm*)	1438.3	1040.0	5560.0	152.3	1094.5	1.12	1.23
	*L/D*	11.2	8.6	51.5	0.8	8.9	1.88	1.35
	*e_t_* (*mm*)	13.0	0.0	300.0	0.0	28.1	25.87	4.08
	*e_b_* (*mm*)	11.2	0.0	300.0	0.0	27.5	29.50	4.38
	*f_y_* (*MPa*)	332.1	322.0	853.0	178.3	81.7	7.83	1.98
	*f_c_* (*MPa*)	46.6	40.1	193.3	7.7	26.8	7.75	2.36
	**Output**							
	*Nexp (kN*)	1616	848.5	46,000	45.2	3181.1	73.86	7.53
**Short**	**Inputs**							
	*D* (*mm*)	169.2	133.1	1020.0	48.0	112.5	23.19	4.17
	*t* (*mm*)	4.2	4.0	13.3	0.5	2.3	1.56	1.15
	*L* (*mm*)	498.7	399.5	3060.0	152.3	334.0	24.32	4.16
	*L/D*	3.0	3.0	4.0	0.8	0.6	0.19	−0.55
	*e_t_* (*mm*)	2.8	0.0	105.0	0.0	10.9	30.38	5.03
	*e_b_* (*mm*)	2.8	0.0	105.0	0.0	10.9	30.38	5.03
	*f_y_* (*MPa*)	336.8	322.7	853.0	185.7	97.5	10.53	2.52
	*f_c_* (*MPa*)	58.8	46.6	193.3	7.7	35.9	2.60	1.54
	**Output**							
	*Nexp* (kN)	2782.5	1678.1	46,000	199.9	4304.5	39.20	5.39

Min. Minimum; Max. Maximum; S.D. standard deviation.

**Table 2:** The setting of different parameters of ANN, ANFIS, and GEP model used in current research.

Parameters	Class and Value	
	*N_lg_*	*N_st_*
Training dataset (70%)	676	495
Testing dataset (30%)	289	207
**ANN**		
Network type	Feed-forward back-propagation	
Data division	Random (un-biased)	
No. of hidden layer	8	
No. of hidden neurons	10	
Training algorithm	Levenberg-Marquardt	
Hidden layer’s Transfer function	TANSIG	
Output layer’s Transfer function	PURELIN	
No. of non-linear parameters	16	
No. of epochs	40	
Learning rate	0.01	
**ANFIS**		
No. of linear parameters	72	65
No. of nonlinear parameters	140	120
Total No. of parameters	176	154
No. of fuzzy rules	5	8
No. of MFs	5	8
No. of nodes	20	45
No. of Training epoch	30	30
Training error goal	0	0
Membership Function type	Trimf	
Fuzzy structure	Sugeno	
Type of FIS	Sub clustering	
Method of Optimization	Back propagation and least square	
Output function	Linear	
**GEP**		
Parameters		
General		
Number of chromosomes	100	
Number of Genes	3	
Head size	8	
Linking function	Addition	
Function set	+, −, ×, ÷	
Numerical constants		
Constant per gene	10	
Type of data	Floating number	
Maximum complexity	8	
Ephemeral random constant	[−10,10]	
Genetic operators		
Rate of mutation	0.00138	
Inversion rate	0.00546	
IS transposition rate		
RIS transposition rate		
One-point recombination rate	0.00277	
Two-point recombination rate		
Gene recombination rate		
Gene transposition rate		

**Table 3:** Statistical indicators for ANN, ANFIS and GEP models developed for bearing capacity of short and long circular CFST columns.

Model	Statistical Metrics	ANN		ANFIS		GEP	
		Training	Testing	Training	Testing	Training	Testing
**Long**	MAE	214.98	196.22	556.86	500.30	306.34	290.36
	MAPE	27.59	25.28	36.29	42.91	40.50	39.74
	RSE	0.0148	0.0086	0.0931	0.0635	0.0256	0.0195
	RMSE	369.15	325.12	925.33	882.60	485.46	489.06
	*R*	0.9929	0.9959	0.9534	0.9678	0.9871	0.9906
	RRMSE	0.2330	0.1922	0.5841	0.5219	0.3064	0.2892
	*PI*	0.1169	0.0963	0.2990	0.2652	0.1542	0.1452
	*OF*	0.1000		0.2700		0.1500	
**Short**	MAE	155.29	145.96	360.05	328.73	350.56	387.93
	MAPE	8.79	9.17	24.55	27.20	22.39	20.15
	RSE	0.00270	0.00274	0.0345	0.0197	0.0179	0.0126
	RMSE	235.77	193.20	750.68	681.67	552.17	527.47
	*R*	0.9986	0.9986	0.9842	0.9907	0.9909	0.9936
	RRMSE	0.0833	0.0273	0.2824	0.2213	0.2023	0.1812
	*PI*	0.0416	0.361	0.1423	0.1111	0.1016	0.0908
	*OF*	0.2300		0.1200		0.090	

**Table 4:** Assessment of GEP model using external testing criterion suggested in the literature.

Equation	Condition	GEP Model	
		Long	Short
$k=\sum_{i=1}^{n}\frac{\left(q_{i}\times p_{i}\right)}{q_{i}{}^{2}}$	0.85 < k < 1.15	0.989	1.00
$k^{\prime }=\sum_{i=1}^{n}\frac{\left(q_{i}\times p_{i}\right)}{p_{i}{}^{2}}$	0.85 < $k^{\prime }$ < 1.15	0.995	0.974
$R_{m}=R^{2}\;(1-\sqrt{\left|R^{2}-R_{0}{}^{2}\right|}$	0.5 < $R_{m}$	0.847	0.877
$R_{0}{}^{2}=1-\frac{\sum_{i=1}^{n}{\left(p_{i}-q_{i}{}^{0}\right)}^{2}}{\sum_{i=1}^{n}{\left(p_{i}-p_{i}{}^{0}\right)}^{2}}$	$R_{0}{}^{2}≅1$	0.999	0.999
${R^{\prime }}_{0}{}^{2}=1-\frac{\sum_{i=1}^{n}{\left(q_{i}-p_{i}{}^{0}\right)}^{2}}{\sum_{i=1}^{n}{\left(q_{i}-q_{i}{}^{0}\right)}^{2}}$	${R^{\prime }}_{0}{}^{2}≅1$	0.979	0.958
$q_{i}{}^{0}=k\times p_{i}$			
$p_{i}{}^{0}=k\times q_{i}$			

## Data Availability

Available on reasonable request from correspondence authors.
